# Using genomics to understand antimicrobial resistance and transmission in *Neisseria gonorrhoeae*

**DOI:** 10.1099/mgen.0.000239

**Published:** 2019-01-30

**Authors:** Leonor Sánchez-Busó, Simon R. Harris

**Affiliations:** Infection Genomics, Wellcome Sanger Institute, Wellcome Genome Campus, Hinxton, Cambridgeshire, UK

**Keywords:** gonorrhoea, genomics, antimicrobial resistance, sexual networks, transmission

## Abstract

Gonorrhoea infections are on the increase and strains that are resistant to all antimicrobials used to treat the disease have been found worldwide. These observations encouraged the World Health Organization to include *Neisseria gonorrhoeae* on their list of high-priority organisms in need of new treatments. Fortunately, concurrent resistance to both antimicrobials used in dual therapy is still rare. The fight against antimicrobial resistance (AMR) must begin from an understanding of how it evolves and spreads in sexual networks. Genome-based analyses have allowed the study of the gonococcal population dynamics and transmission, giving a novel perspective on AMR gonorrhoea. Here, we will review past, present and future treatment options for gonorrhoea and explain how genomics is helping to increase our understanding of the changing AMR and transmission landscape. This article contains data hosted by Microreact.

## Data Summary

1. Metadata for the genomic epidemiology studies reviewed in this work can be found in the respective citations (see data bibliography).

**Impact Statement**

The rise of gonococcal strains that are resistant to last-line antimicrobial treatment options endangers the long-term control of gonorrhoea. To address this, new antimicrobials and combination therapies are being developed and tested. To tackle antimicrobial-resistant gonorrhoea, we first need to understand how it evolves resistance and how it is spread in sexual networks. In this review, we provide an overview of how genomic analyses are being used to provide insight in these areas.

## Introduction

The diplococcoid, betaproteobacterium *Neisseria gonorrhoeae* is the causative agent of gonorrhoea, one of the most common sexually transmitted infections (STIs) worldwide. Nearly 80 million cases are recorded each year with 45 % of those in the Western Pacific region alone [1]. Although gonorrhoea infection is rarely fatal, the burden of disease is high [2], particularly among men. However, women are more often subject to problematic infections due to higher rates of chronic infection. This is because urogenital infection is initially asymptomatic in more than 50 % of women but only 10 % of men [3], leading to missed opportunities for treatment. Complications include damage to the upper genital tract such as pelvic inflammatory disease in women or more rarely epididymitis in men, which can lead to reproductive problems and even infertility [3]. High-risk populations for gonorrhoea infection include sexual networks that partake in unprotected sex with multiple partners, particularly commercial sex workers, men who have sex with men (MSM) and young heterosexuals [4, 5], although recent work suggests that it is the frequency of antimicrobial treatment, and not the number of sexual partners, which really contributes to its successful spread [6]. Here, we will review the past and novel potential treatment options for gonorrhoea infections as well as illustrate how whole-genome sequencing (WGS) has provided insight into the population and transmission dynamics of *N. gonorrhoeae* within different sexual networks.

## Rapid Acquisition of Antimicrobial Resistance

Various antimicrobials have been used to treat gonorrhoea over the past century. Since the introduction of sulfonamides in the 1930s, penicillins, tetracyclines, fluoroquinolones, macrolides and cephalosporins have all been used to treat the disease [3]. According to recommendations of the World Health Organization (WHO), a treatment stops being recommended when the prevalence of resistance is over 5 % [7], which has already occurred for many antimicrobials, in some cases very rapidly after their introduction [8]. The fluoroquinolone ciprofloxacin was very widely used to treat gonorrhoea in the 1980s, but resistant cases appeared during the following decade and in 2007 it was removed as first-line treatment [8]. This was replaced with the oral extended-spectrum cephalosporin (ESC) cefixime, which was itself deemed to be failing in 2011 due to global reports of resistance following an initial case described in Japan [9]. The current recommended treatment in many countries is a dual therapy of the macrolide azithromycin and the ESC ceftriaxone [10]. Strains harbouring resistance to either ceftriaxone or azithromycin are not uncommon, however. Since 2009, ceftriaxone-resistant strains have been found in Japan [11], France [12], Spain [13], Australia [14] and very recently Canada [15]. Azithromycin resistance is also on the increase [16], with high-level resistant [minimum inhibitory concentration (MIC)≥256 mg l^−1^] isolates causing sporadic infections in several parts of the world [17–21] and even an ongoing outbreak that started in 2014 in Leeds, UK [22]. Interestingly, ceftriaxone is also used to treat diseases of the urinary tract and azithromycin monotherapy is often used to treat other STIs, such as *Chlamydia trachomatis* infection. In fact, its use to treat parallel co-infections with other STIs has been hypothesized to play a role in the increase of resistance to azithromycin in gonorrhoea infections [16]. Fortunately, concurrent resistance to both antimicrobials in the dual therapy is rare [23]. In fact, it was recently observed that coexistence of the genetic determinants that cause azithromycin and ESC resistance appear to confer a fitness decrease, although further work is required in this area [24]. The existence of *N. gonorrhoeae* strains that are resistant to even the last-line antimicrobials and the fear of not having a suitable alternative to treat the disease led the WHO to include gonorrhoea on its 2017 list of ‘high-priority’ pathogens for which there is a need for research and development of new antimicrobials [25].

## Genomic Insight into AMR Gonococcus

**Fig. 1.**
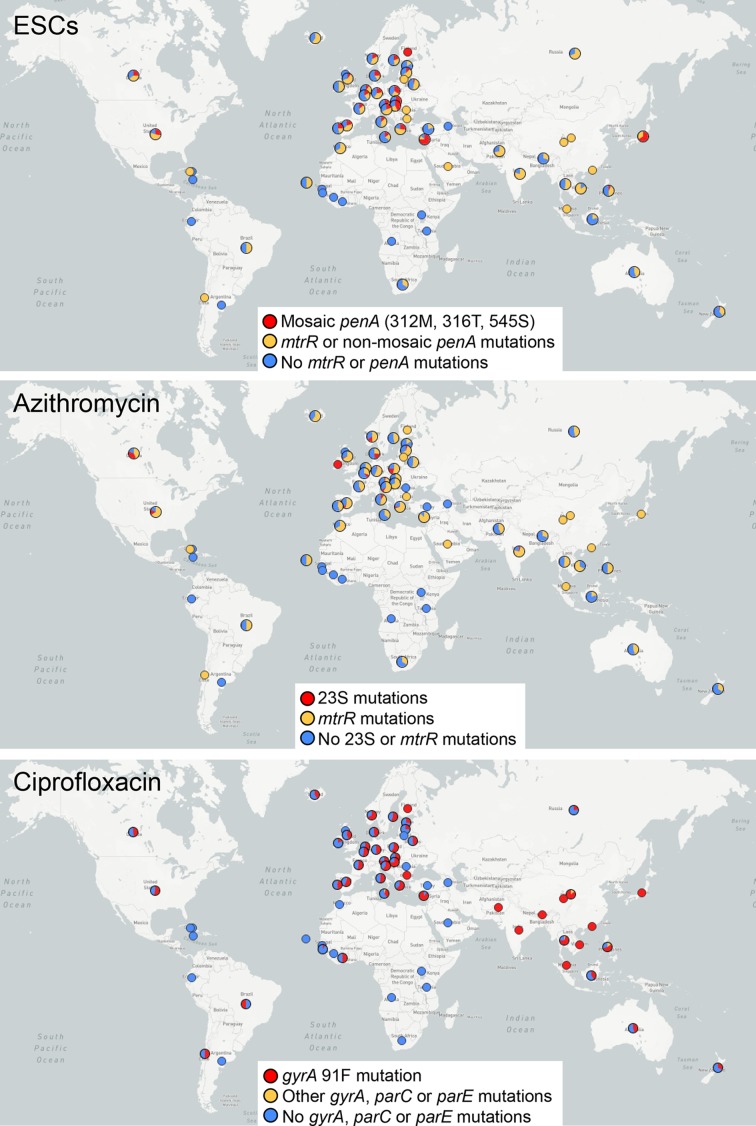
Distribution of AMR determinants on *N. gonorrhoeae* isolates from genome-based published papers. The most relevant known mutations [3] were tested from raw WGS data using ARIBA [53]. Those reported to produce the highest increase in MIC [29, 34] are coloured in red, while other associated mutations reported to cause a reduced susceptibility phenotype are coloured in orange and no known mutation in blue. Some mutations have not been proven to cause an MIC increase above the breakpoints. Note that some publications used a biased sampling to fulfil their particular work aims, so the maps are not fully representative of the incidence of the resistance mutations in particular locations. Maps were obtained using Microreact [54], and a dynamic version that includes the individual tested determinants can be found at https://microreact.org/project/rJQ6FGj8G. Grey represents isolates with missing metadata.

WGS has provided further insight into antimicrobial resistance (AMR) in the gonococcus, and several genetic determinants have been found associated with decreased susceptibility and resistance to key antimicrobials [8]. In addition, WGS of large collections of isolates around the world has revealed details on the evolution of AMR, but also on gonorrhoea population structure, intercontinental spread and transmission in different risk groups (Table 1). A compilation of the geographical source and genotypic AMR of the genomes of *N. gonorrhoeae* isolates sequenced to date is shown in Fig. 1.

**Table 1.** List of publications that use WGS to study the population dynamics and distribution of antimicrobial resistance in different parts of the world

Regions covered	Publication	Ref.	Main aim	Main findings
Worldwide	Ezewudo *et al.* 2015	[55]	Study the population structure, dynamics and the evolution of antimicrobial resistance in *N. gonorrhoeae*.	Population grouped into five clusters, involved in a considerable level of recombination and two of them harbouring mostly resistant strains to azithromycin and cefixime.
	Sánchez-Busó *et al.* 2018	[29]	Study when and where modern gonococcal populations (1960–2013) emerged and what has been the impact of antimicrobial usage and transmission in different sexual networks.	Modern gonococcus was estimated to have emerged in the 16th century in Africa or Europe. AMR determinants probably emerged in Asia and spread worldwide. The existence of two (multi-resistant and multi-susceptible) lineages of *N. gonorrhoeae* with different AMR patterns, different recombination rates and adapted to different sexual networks with variable chance of co-infection and treatment.
Canada	Demczuk *et al.* 2015	[30]	Study the genomic variability of *N. gonorrhoeae* with decreased susceptibility to cephalosporins in Canada (1989–2013).	Canadian gonococcal population grouped into 12 clusters. Decreased susceptibility to ESCs emerged during the 1990s followed by the introduction of *penA* mosaics X and XXXIV.
	Demczuk *et al.* 2016	[31]	Study the genomic epidemiology and AMR mechanisms of azithromycin-resistant *N. gonorrhoeae* in Canada (1997–2014).	Geographical and temporal clustering indicated multiple independent acquisitions of azithromycin resistance, with a subsequent rapid clonal expansion through local sexual networks.
USA	Grad *et al.* 2014	[26]	Study the genomic epidemiology of *N. gonorrhoeae* strains with reduced susceptibility to cefixime in the USA (2009–2010) to reconstruct the likely spread of lineages through different sexual networks.	Mosaic *penA* XXXIV is significantly linked to cefixime resistance. A lineage of strains harbouring reduced susceptibility to this antibiotic was observed to have spread eastward, including several cases of transmission from MSM to heterosexual populations.
	Grad *et al.* 2016	[24]	Study the genomic epidemiology of *N. gonorrhoeae* strains resistant to cephalosporins, macrolides and fluoroquinolones in the USA (2000–2013).	Reduced susceptibility to ESCs is mostly clonal and associated with *penA* XXXIV, while azithromycin resistance has arisen through several mechanisms and showed limited clonality. Fluoroquinolone resistance has also arisen multiple times and extensively spread in a clonal manner.
Europe	Jacobsson *et al.* 2016	[16]	Study the genomic epidemiology of azithromycin-resistant (MIC>2 mg l^−1^) *N. gonorrhoeae* in Europe (2009–2014).	Reduced number of azithromycin-resistant strains spread clonally. Two previously described 23S rRNA mutations explained most of the observed resistance.
	Harris *et al.* 2018	[34]	Use WGS to analyse a European survey of *N. gonorrhoeae* conducted during 2013 in terms of genomic epidemiology and AMR	Genogroup G1407 was predominant and accounted for most cases of cephalosporin resistance, although it reduced since the previous survey in 2009–2010. Also, the association of G1407 to MSMs changed to heterosexuals in the survey presented in this study.
UK	Didelot *et al.* 2016	[33]	Study two local collections of isolates from the UK: one from Sheffield collected over 6 years from a mostly heterosexual population, and another one from London during 6 months mostly associated with MSMs.	The Sheffield set showed transmission was associated with a median time to the most recent common ancestor of about 3 months. This threshold applied to the London dataset revealed transmission occurring among cases of similar age, sexual orientation, location and human immunodeficiency virus (HIV) serostatus.
	Chisholm *et al.* 2016	[22]	Use WGS to analyse eight strains with high-level azithromycin resistance from Leeds (2014–2015) in the context of other UK cases.	An outbreak was confirmed using epidemiological, microbiological and also genomic information.
	De Silva *et al.* 2016	[32]	Use WGS to study transmission among patients attending sexual health clinics in Brighton (2011–2015) including intercontinental transmissions (other UK sites and USA).	Multiple samples were observed to be related across geographical locations. A transmission nomogram was described that can be used to determine direct or indirect transmission between two cases using genetic data and the time between both cases. Most of the detected clusters with >10 patients comprised only men.
New Zealand	Lee *et al.* 2017	[39]	Study the genomic epidemiology and antimicrobial resistance of *N. gonorrhoeae* in New Zealand (2014–2015).	Eleven clusters were identified, with decreased susceptibility to ESCs in only 3.5 % of isolates. Clusters contained a high proportion of females, suggesting transmission in New Zealand does not occur exclusively among MSM.

Genomic epidemiology has been used to study the emergence, evolution and spread of the gonococcus in different parts of the world, with particular focus on AMR strains. Grad *et al.* [26] were the pioneers in studying the genomic epidemiology of AMR gonococcus in the USA, first with isolates with reduced susceptibility to cefixime, helping to understand its transmission within the USA. Following previous observations of mosaic *penA* alleles linked to reduced susceptibility to ESCs [27], they found a strong association between resistance and the presence of the mosaic *penA* XXXIV allele in two different lineages. These mosaic *penA* alleles are known to be formed via recombination with other *Neisseria* species [28]. A later, broader study on fluoroquinolone-, macrolide- and ESC-resistant gonococcus in the USA by the same group [24] revealed novel loci that may be involved in reducing susceptibility to ceftriaxone and azithromycin. In contrast, fluoroquinolone resistance could be explained in >95 % of cases using only one genomic marker *(gyrA* 91F) [29]. Decreased susceptibility to ESCs and azithromycin has also been examined using genomic epidemiology in Canada by Demczuk *et al*. [30, 31]. Their results showed multiple independent acquisitions of ESC and azithromycin resistance over time followed by clonal expansion within high-risk sexual networks [31]. In Europe, *N. gonorrhoeae* AMR has been shown to follow the same pattern [16, 32, 33]. However, a recent analysis of isolates from a 2013 ECDC Euro-GASP survey in 20 European countries [34] revealed a decrease in the ESC-resistant genogroup G1407 from a 2011 survey, traditionally linked to MSM populations but associated with heterosexuals in 2013. As part of the 2013 survey, the genomics used to assess the two main typing schemes, multilocus sequence typing (MLST) and *N. gonorrhoeae* multi-antigen sequence typing (NG-MAST), showed that they do not provide enough resolution to confidently identify isolates from particular lineages, yet can often erroneously cluster highly divergent lineages due to the considerable level of recombination in the gonococcus.

WGS provides a level of discrimination among strains that allows analysis down to the level of transmission clusters. Grad *et al.* [26] detected introductions from MSMs into the heterosexual population in the USA using WGS, while two other studies focused on gonorrhoea transmission in different regions and sexual networks in the UK. Both highlighted the importance of combining genomic data with contact tracing and epidemiological information to more accurately infer transmission [32, 33]. Didelot *et al.* analysed a set of NG-MAST sequence type 12 (ST12) isolates from a single clinic collected over 6 years in a mostly heterosexual population in Sheffield, UK, for which contacts reported by patients were known. They were able to show that sexual contacts between cases of transmission occurred a mean of 3.4 months before sampling. Applying this threshold to another collection conducted over 6 months in several clinics in London, UK, without using contact tracing information, predicted transmission links that correlated accurately with the available epidemiological information [33]. Transmission was also investigated by De Silva *et al.* in a large collection of isolates from a single year from Brighton, UK. They developed a new tool called a ‘nomogram’ to predict transmission links by comparing the number of SNPs between the infecting isolates with the time between sampling dates [32]. Their results showed that over 70 % of the isolates sampled within 3 months were expected to be linked either by direct or by indirect transmission. Further work on this dataset showed that phenotypic resistance in gonorrhoea could be accurately predicted from the known AMR genomic determinants using WGS [35].

Gonorrhoea with novel AMR phenotypes has often been first reported in Asia. Genomic data have provided a possible explanation for this, identifying that an AMR-associated lineage, which emerged in Asia, has subsequently repeatedly disseminated globally [29]. Genetic analysis of target genes combined with phenotypic information conducted by Shimuta *et al*. [36] revealed the first cefixime-resistant isolate containing a mosaic *penA* X allele in 1997 in Japan, although a previous isolate with reduced susceptibility and without genotypic information had already been detected in 1995. These two instances of cefixime resistance were followed by an increase in the number of isolates with reduced susceptibility over time in Japan and subsequently globally [36]. In the period from 2000 to 2015, 5–10 % of Japanese isolates were resistant to at least one of the antimicrobials in the current dual therapy [37]. In the same period, China also reported an increasing number of isolates with decreased susceptibility to ceftriaxone and at least one with an azithromycin MIC of 1 mg l^−1^ [38]. The only genomic analysis from the West Pacific region came from a recent analysis by Lee *et al*. [39], who looked at the gonococcal population in New Zealand. Their study revealed highly clonal clusters with a significant proportion of isolates coming from females, suggesting the sexual networks driving gonorrhoea transmission in this region are less dominated by MSM, but instead were mostly made up by a combination of MSM with heterosexual or bisexual networks. Very few isolates with decreased susceptibility to ESCs were found, some of them with *penA* XXXIV as an associated genetic determinant. Only two azithromycin-resistant isolates contained the low-level 23S mutation (C2611T), although more than 10 % of all the isolates revealed a reduced susceptibility due to mutations in *mtrR* or its promoter.

On a global scale, Sánchez-Busó and colleagues revealed the population structure of a broader globally isolated set of *N. gonorrhoeae* isolates from the 1960s until 2013 [29]. Interestingly, genome-based phylogenetics revealed the probable existence of two lineages of the pathogen, one of them associated with a higher recombination rate, harbouring more AMR determinants and probably associated with high-risk groups that are more often subjected to antimicrobial treatment for gonorrhoea and other STIs. By contrast, the second lineage was associated with a lower recombination rate, fewer AMR determinants and networks which may be subjected to higher rates of asymptomatic infections, particularly in women. The 2013 European survey of gonorrhoea infections [34] clearly supports this observation, showing that the multi-susceptible lineage is significantly associated with women and heterosexual populations in general. Further studies will be needed to characterize this lineage, which is potentially silently spreading worldwide.

## New Antimicrobials and Point-of-Care Testing

A suitable vaccine to prevent gonococcal infections is currently not available. Previous attempts to develop a vaccine have not produced sufficient protection because the targeted gonococcal surface antigens are too rapidly evolving, and there is a lack of a suitable animal model for systematic testing of protective responses on a larger panel of antigens [40]. Interestingly, a recent study in New Zealand suggested that the group B meningococcal outer membrane vesicle vaccine could reduce the risk of gonorrhoea infection [41]. This provides hope that comparative genomics can support the development of novel vaccination strategies by providing data on antigenic variation and immune selection in circulating strains [42]. In the absence of a suitable vaccine, there are ongoing efforts to find alternative antimicrobials. A phase 3 randomized controlled trial (RCT) is evaluating the effectiveness, tolerability and safety of solithromycin as an azithromycin [43] or ceftriaxone [44] replacement. Zoliflodacin and gepotidacin are other antimicrobials under clinical evaluation for the treatment of gonococcal infections [45, 46]. Alternatively, a non-inferiority phase 3 RCT is assessing intramuscular gentamicin plus oral azithromycin for uncomplicated anogenital and pharyngeal gonorrhoea [47]. Spectinomycin is being re-evaluated due to the high level of global susceptibility, although there is fear that resistances could be rapidly selected unless it is included in a dual therapy with, for example, solithromycin [45]. Several other new antimicrobials have also proven efficient *in vitro* [45, 48], such as the fluoroquinolone sitafloxacin [49]. One important consideration is that treatment should potentially be adjusted to the type of infection, as pharyngeal infections are more difficult to treat than urogenital infections [50]. Tuite and colleagues [51] highlighted the importance of a rapid point-of-care (POC) test for personalized diagnosis, and showed that the current non-individualized treatment could result in >5 % strains being resistant to the current dual treatment in the next 15 years, while the use of a POC test could delay this event. POC tests could additionally detect susceptibilities to antimicrobials that are no longer recommended, e.g. fluoroquinolones, and thus allow treatment of individual infections without contributing to increased pressure on the last-line treatments. The increasing availability of genomic data has the potential to enable development of POC tests and could inform on resistance pathways to these new antimicrobials. Current tools used for pathogen surveillance and resistance prediction, such as WGSA (www.wgsa.net), Mykrobe [52], ARIBA [53] and several others, can be used in this development process as well as for surveillance of AMR strains.

## Conclusion

AMR tracking is of utmost importance for the control of gonorrhoea and to avoid reaching a point where the disease becomes untreatable. Resistance to the last-line antimicrobials, especially azithromycin, is occurring globally, although resistance to dual therapy is still rare. Fortunately, genomics is a step change in the fight against AMR, allowing detection of known genotypic determinants and the discovery of novel ones. This also allows detection of discordances between resistance phenotypes and genotypes that was difficult using traditional targeted molecular amplification. Furthermore, genomic studies have unveiled the population structure and dynamics of this sexually transmitted pathogen and have provided insight into how it is being transmitted in different networks. Having a resolved population structure allows reconstruction of events from the fine scale, i.e. transmission, up to the very large scale, such as the recognition that there are two main lineages in the current population that diverged hundreds of years ago. The multi-resistant lineage, mostly spread in high-risk groups, has been the main focus for WGS studies to date. However, just as importantly, a second, multi-susceptible lineage seems to be silently spreading in lower-risk, potentially undertreated groups, and although they do not pose a challenge for AMR, they do pose a public health risk, and further work to survey the behaviour of this lineage and potential interaction with AMR strains may be necessary.

## Data bibliography

1. Grad YH *et al*. https://doi.org/10.1016/S1473-3099(13)70693-5 (2014).
2. Ezewudo MN *et al*. https://doi.org/10.7717/peerj.806 (2015).
3. Demczuk W *et al*. http://dx.doi.org/10.1128/JCM.02589-14 (2015).
4. Demczuk W *et al*. http://dx.doi.org/10.1128/JCM.03195-15 (2016).
5. Grad YH *et al*. http://dx.doi.org/10.1093/infdis/jiw420 (2016).
6. Jacobsson S *et al*. http://dx.doi.org/10.1093/jac/dkw279 (2016).
7. Chisholm SA *et al*. http://dx.doi.org/10.1136/sextrans-2015-052312 (2016).
8. Didelot X *et al*. http://dx.doi.org/10.1128/mBio.00525-16 (2016).
9. Eyre DW *et al*. https://doi.org/10.1093/jac/dkx067 (2017).
10. Lee RS *et al*. http://dx.doi.org/10.1093/jac/dkx405 (2017).
11. Harris SR *et al*. https://doi.org/10.1016/S1473-3099(18)30225-1 (2018).
12. Sánchez-Busó L *et al*. https://doi.org/10.1101/334847 (2018).
